# Two-Tier Efficient QoE Optimization for Partitioning and Resource Allocation in UAV-Assisted MEC †

**DOI:** 10.3390/s24144608

**Published:** 2024-07-16

**Authors:** Huaiwen He, Xiangdong Yang, Feng Huang, Hong Shen

**Affiliations:** 1 School of Computer, Zhongshan Institute, University of Electronic Science and Technology of China, Zhongshan 528402, China; yangxiangdong@std.uestc.edu.cn (X.Y.); 202321080835@std.uestc.edu.cn (F.H.); 2 School of Computer Science and Engineering, University of Electronic Science and Technology of China, Chengdu 611731, China; 3 School of Engineering and Technology, Central Queensland University, Rockhampton 4701, Australia; h.shen@cqu.edu.au

**Keywords:** unmanned aerial vehicle, multi-access edge computing, task offloading, large-scale IoT network, shrinkage ratio

## Abstract

Unmanned aerial vehicles (UAVs) have increasingly become integral to multi-access edge computing (MEC) due to their flexibility and cost-effectiveness, especially in the B5G and 6G eras. This paper aims to enhance the quality of experience (QoE) in large-scale UAV-MEC networks by minimizing the shrinkage ratio through optimal decision-making in computation mode selection for each user device (UD), UAV flight trajectory, bandwidth allocation, and computing resource allocation at edge servers. However, the interdependencies among UAV trajectory, binary task offloading mode, and computing/network resource allocation across numerous IoT nodes pose significant challenges. To address these challenges, we formulate the shrinkage ratio minimization problem as a mixed-integer nonlinear programming (MINLP) problem and propose a two-tier optimization strategy. To reduce the scale of the optimization problem, we first design a low-complexity UAV partition coverage algorithm based on the Welzl method and determine the UAV flight trajectory by solving a traveling salesman problem (TSP). Subsequently, we develop a coordinate descent (CD)-based method and an alternating direction method of multipliers (ADMM)-based method for network bandwidth and computing resource allocation in the MEC system. Extensive simulations demonstrate that the CD-based method is simple to implement and highly efficient in large-scale UAV-MEC networks, reducing the time complexity by three orders of magnitude compared to convex optimization methods. Meanwhile, the ADMM-based joint optimization method achieves approximately an 8% reduction in shrinkage ratio optimization compared to baseline methods.

## 1. Introduction

In recent years, mobile edge computing (MEC) has emerged as a prominent computing paradigm to accommodate the rapid proliferation of internet of things (IoT) devices and novel application scenarios such as virtual reality (VR), augmented reality (AR), autonomous driving, and intelligent robotics [1]. However, ground-based MEC networks face inherent limitations in coverage, deployment flexibility, and the handling of hotspot issues due to the immobility of MEC servers. The limitations of stationary infrastructure present challenges in accommodating the dynamic edge network environment. Consequently, researchers have increasingly focused on unmanned aerial vehicle (UAV)-assisted MEC (UAV-MEC), which offers significant advantages in terms of high mobility and low deployment costs [2,3].

The existing body of work addresses numerous challenges associated with UAV-MEC systems, including the limited computational and battery capacities of UAVs, trajectory planning, cooperative control of multiple UAVs, and data transmission security. Various optimization problems have been formulated to tackle these challenges by adjusting offloading decisions, bandwidth allocation, caching strategies, and UAV trajectories. Common optimization objectives include minimizing task execution time [4], reducing energy consumption [5,6], or balancing a weighted combination of both [7]. For instance, resource allocation and task offloading ratios are jointly optimized to minimize the total energy consumption of user devices (UDs) under partial offloading strategies [8], and the joint design of UAV trajectory, task allocation, and communication resource management aims to minimize a weighted sum of execution latency and energy consumption [9]. Similarly, maximizing computational efficiency to ensure robust system performance is another focus [10,11]. However, these objectives often emphasize system performance over the quality of experience (QoE) for UDs, potentially leading to unfair resource allocation.

From the users’ perspective, it is crucial to consider both waiting time and execution time to measure QoE in UAV-MEC systems [12]. Several studies have addressed this by formulating optimization problems aimed at minimizing task response time, which encompasses both waiting and execution times [13,14]. For instance, [15] proposes a joint optimization approach that considers offloading ratios, service policies, and UAV trajectories to minimize the maximum delay of delay-sensitive tasks in each time slot. Furthermore, [16] introduces a comprehensive metric called the shrinkage ratio, balancing factors such as waiting time, execution time, task length, and UD computational capabilities, providing a straightforward and effective measure of optimization efficiency in UAV-MEC networks. However, these studies often assume that computation tasks are divisible and do not fully account for the impact of a large number of UDs in UAV-MEC systems. In reality, the increasing number of UDs and the binary offloading model for indivisible tasks present significant challenges in maintaining good QoE for users.

This paper investigates the QoE-oriented computation task binary offloading optimization problem in a large-scale UAV-MEC network, which may involve hundreds or thousands of IoT devices. Our goal is to minimize the total shrinkage ratio across all UDs by jointly optimizing UAV trajectory, task offloading mode selection, network bandwidth allocation, and computation resource allocation. To handle the high complexity arising from the large number of UDs and the coupling of drone trajectory and resource allocation for computation tasks, we propose an efficient two-tier optimization scheme. We first develop a low-complexity partition coverage algorithm using the Welzl method to determine the UAV flight trajectory by solving a traveling salesman problem (TSP). Following this, for the sub-problem of network bandwidth and computing resource allocation, we introduce two methods. For large-scale networks where algorithm efficiency is paramount, we develop a low-complexity CD-based method. In contrast, for scenarios that prioritize optimization efficiency and have more flexible timeliness requirements, we design a joint optimization method based on the ADMM technique.

The main contributions of this paper are summarized as follows:

1. We extend the model presented in [16] to address binary task offloading in UAV-MEC networks that encompass a large-scale node deployment, potentially numbering in the hundreds or thousands. This extension introduces a more complex, tightly coupled MINLP problem due to the binary offloading decisions and the quality of experience (QoE)-oriented optimization objectives. Concurrently, the substantial number of edge nodes presents significant challenges to the operational efficiency of conventional numerical optimization techniques.
2. We introduce a two-tier optimization scheme that effectively decouples UAV trajectory planning from computation task resource allocation. In the initial tier, we implement partitioning to diminish the problem’s complexity, transitioning from a large-scale node scenario to a series of more manageable small-scale partitions. We propose a circle-covering algorithm inspired by the Welzl method to adeptly address the UAV set-covering problem. This approach allows the UAV’s trajectory to be contingent upon these partitions rather than the precise locations of each node, significantly streamlining the path planning process.
3. We develop CD-based and ADMM-based methods for task offloading mode selection and resource allocation in the second tier. The CD-based method has a linear complexity concerning the network scale, while the ADMM-based approach converges rapidly and achieves a lower shrinkage ratio compared to alternating optimization methods.
4. We conduct extensive numerical simulations to thoroughly evaluate the effectiveness and practicability of our proposed algorithms. The CD-based method demonstrates exceptional efficiency, outperforming other benchmark methods by reducing the time complexity by three orders of magnitude and achieving a 5% reduction in the shrinkage ratio. In comparison to traditional alternating optimization methods, our proposed ADMM-based method requires 50% fewer iterations for convergence and realizes an approximate 8% improvement in shrinkage ratio optimization.

The rest of this paper is organized as follows: Section 2 discusses the related work on UAV-MEC. In Section 3, we present a system model for user QoE optimization in large-scale edge networks and formulate it as a combinatorial optimization problem. Section 4 provides the algorithm for solving this optimization problem. In Section 5, we validate the effectiveness of the algorithm through numerical experiments. Section 6 concludes this paper and discusses future work.

## 2. Related Work

### 2.1. Task Offloading in UAV-MEC

UAVs have become a focal point in the realm of MEC, offering notable advantages such as reduced cost and enhanced flexibility. These attributes significantly bolster the coverage and efficiency of edge networks, as illustrated in [3]. The optimization of 3-D multi-UAV trajectories, as examined by He et al. [17], ensures equitable task distribution and curtailed energy usage. Zhang et al. [18] have leveraged UAVs as aerial relays, facilitating signal transmission to edge servers and devices from both ground and air. Liu et al. [5] delve into UAVs’ role as computationally limited MEC nodes, extending processing power to terminal devices and aiding in task offloading for locally infeasible computations. Gao et al. [19] tackle secure data transmission in UAV-assisted maritime MEC systems, proposing an optimization strategy for transmit power, time slot allocation, and UAV trajectory to fortify system security and computational performance.

Furthermore, the convergence of UAVs with cutting-edge communication technologies is a subject of ongoing research. Jiao et al. [20] concentrate on enhancing the data rate for strong signal users in IRS-aided UAV-NOMA (non-orthogonal multiple access) networks while ensuring the requisite data rate for weaker signals by optimizing the positioning of intelligent reflecting surface (IRS) and UAVs. Wang et al. [21] present an energy-efficient scheme for UAV-MEC, targeting traffic offloading in Terahertz (THz) frequency bands. In the context of 6G networks, M. A. Baker Siddiki Abir et al. [22] introduce a pioneering digital twin-based aerial MEC framework, harnessing UAVs as aerial base stations equipped with MEC functionalities to ensure superior network performance for real-time and latency-sensitive services. However, the majority of existing works primarily focus on performance metrics of the UAV-MEC systems, such as energy consumption and data processing rates, with less emphasis on the user-centric QoE indicators that are critical to end-user satisfaction and system usability.

### 2.2. QoE-Oriented Optimization

QoE is a pivotal metric in UAV-MEC systems, capturing the effectiveness of optimization efforts and garnering considerable research interest. Shen et al. [16] have introduced the shrinkage ratio, a novel metric that quantifies the optimization efficiency delivered to users by the UAV-MEC network. This concept reshapes QoE optimization as a targeted minimization of the shrinkage ratio. Expanding on this, Liu et al. [12] examined the data aggregation challenge within a multi-layer UAV-MEC system, comprising a central node and UAVs with distinct responsibilities. They developed a service satisfaction model tailored for UAVs and employed a low-complexity matching game approach to achieve UAV node selection and resource allocation. Furthermore, Tian et al. [23] proposed a three-tier UAV-MEC network architecture encompassing users, drones, and a cloud center. They introduced the response ratio, an optimization metric grounded in user preferences and real-time demands, aiming to enhance service delivery and system responsiveness.

### 2.3. Optimization Approaches in UAV-MEC Networks

Resource allocation and task offloading in UAV-MEC networks are commonly addressed through numerical optimization methods or metaheuristic strategies. Chen et al. [15] crafted an efficient two-tier optimization framework leveraging metaheuristic techniques; the first tier integrates particle swarm optimization (PSO) with genetic algorithms (GA) for UAV deployment, while the second tier utilizes a greedy algorithm for computation offloading. Tun et al. [24] incorporated communication and computation delays into constraints and explored the energy consumption minimization problem, introducing a block successive upper-bound minimization (BSUM) algorithm to address the resulting non-convex challenges. Zeng et al. [25] focused on dynamic data collection via UAV-mounted sensors, employing a hybrid algorithm combining block coordinate descent (BCD) with successive convex approximation (SCA) to mitigate the non-convex energy consumption minimization issue. Xu et al. [26] developed a scalable optimization framework that integrates graph theory and convex optimization, effectively managing UAV trajectories and resource allocation in dense urban settings, thereby reducing computational complexity and catering to the diverse needs of heterogeneous IoT devices. However, these methods can encounter high complexity without tailored optimization strategies.

### 2.4. Learning-Based Algorithms in UAV-MEC Networks

The integration of learning algorithms, particularly deep reinforcement learning (DRL), has emerged as a potent solution for dynamic resource allocation and online task offloading in UAV-MEC networks. Xiang et al. [27] harnessed a combination of differential evolution (DE) and optimistic actor-critic (OAC) algorithms to refine task offloading decisions and UAV trajectories. This dual approach effectively minimizes a weighted sum of system energy consumption and latency. Liu et al. [28] implemented DRL to dynamically optimize UAV trajectories and resource allocation, thereby enhancing system efficiency in fluctuating network scenarios. Chen et al. [29] tackled the time-sequential MINLP problem in multi-UAV-assisted task offloading by employing DRL to strategize UAV trajectories. They complemented this with the derivation of optimal closed-form solutions for transmission power and computational resources, leading to efficient and effective solutions. Hou et al. [30] introduced an innovative covert federated learning architecture utilizing UAVs. These UAVs play a dual role, orchestrating federated learning operations while generating artificial noise to deter eavesdropping by unauthorized entities. Li et al. [31] explored the application of federated learning (FL) in UAV-assisted MEC, facilitating distributed model training that prioritizes data privacy—a critical feature for applications involving sensitive data.

Different from the aforementioned work, we propose a binary task offloading model aimed at enhancing user QoE in large-scale edge networks, a realm where high-complexity algorithms’ inefficiencies become apparent. Our research diverges from existing works by focusing on two aspects: (1) Firstly, we tackle the scalability challenge inherent in large-scale networks comprising hundreds to thousands of nodes, where the inadequacy of current algorithms is significantly magnified. (2) Secondly, we address the non-differentiability in binary offloading for shrinkage ratio optimization, an issue that renders traditional gradient-based optimization techniques inapplicable. Our approach circumvents these issues through partition-based optimization objectives coupled with a strategic blend of alternating and joint optimization to achieve both efficiency and solution quality.

## 3. System Model and Problem Formulation

**Figure 1:**
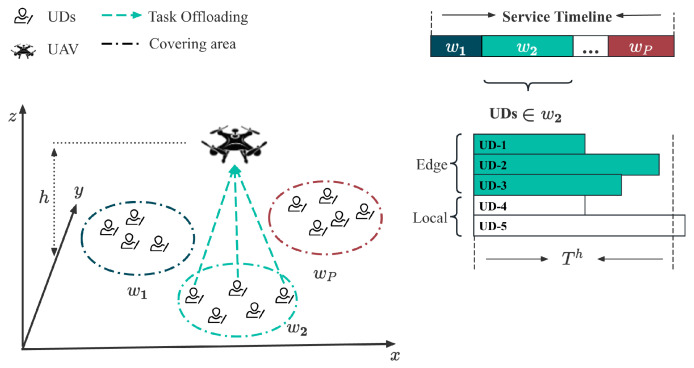
Architecture of the UAV-MEC network with Hover-Fly Mode.

We consider the UAV-assisted MEC network as shown in Figure 1, which comprises *I* UDs and a UAV equipped with a lightweight edge computing server. The set of all UDs is denoted by I={1,2,…,I}. Each UD has an indivisible, computation-intensive task $o_{i}$ characterized by the tuple $\langle c_{i},u_{i}\rangle$ within a time period, where $c_{i}$ represents the amount of computation workload (in CPU cycles), and $u_{i}$ denotes the data size (in bytes). It is assumed that each generated task can be completely processed in a single time period.

### 3.1. UAV Coverage Partition and Flight Model

In a reality IoT network, the spatial distribution of UDs usually exhibits a certain level of clustering [32], so here we partition them into multiple circles with the same radius, which equals the coverage radius of UAV [33]. Let P={1,2,…,P} denote all the coverage circles of UAV, where $q_{p}=\left(x_{p},y_{p},0\right)$ represents the coordinates of the center of coverage circle *p*, and the set $I_{p}$ represents all user equipment located within coverage circle *p*. Let $q_{m}=\left(x_{m},y_{m},0\right)$ denote the location of UD-*m*, where $x_{m}$ and $y_{m}$ represent the horizontal and vertical coordinates, satisfying $0\leq x_{m}\leq x^{\max}$, $0\leq y_{m}\leq y^{\max}$, ∀m.

We assume that UAVs employ a hover-fly-hover mode similar to [34]. The UAV’s movement can be simplified to constant-speed linear motion between multiple coverage circles. The UAV does not perform task offloading while in transit and only provides task computing services to UDs within a coverage circle when hovering above it. The UAV starts in a designated coverage area and traverses all coverage circles along the shortest path. Let the non-repetitive ordered set $w=\{w_{1},w_{2},\ldots ,w_{P}|w_{p}\in P,\forall p=1,2,\ldots ,P\}$ represent the UAV’s trajectory. We assume that the UAV’s battery is sufficient to support its flight, hovering, and mission computation, aligning with the approach in [35].

### 3.2. Computation Model

Due to the indivisibility of computation tasks, we adopt a binary task offloading mode for each UD. We define a binary decision variable $d_{i}\in \left\{0,1\right\}$ for UD *i* to determine the offloading choice. If $d_{i}$ equals 1, the computation task is offloaded to the edge server on the UAV; otherwise, the task is processed locally.

#### 3.2.1. Local Computing

If $d_{i}=0$, then UD *i* can process the computation task locally at the initial stage of the system without waiting for the UAV’s arrival. Let $f_{i}^{l}$ denote the local CPU frequency of UD *i*. Thus, the total delay of task $o_{i}$ in local computation mode can be expressed as:

(1) $$\begin{array}{cc} \; & T_{i}^{l}=\frac{c_{i}}{f_{i}^{l}} \end{array}$$

#### 3.2.2. Edge Computing

If $d_{i}=1$, then UD *i* needs to wait for the UAV to reach its partition and then offload tasks to the UAV for execution. When the UAV is hovering over coverage circle *p*, multiple user devices within the set $I_{p}$ communicate with the UAV through orthogonal frequency-division multiple access (OFDMA) such that interference among multiple UDs can be neglected. During the task offloading process, the uplink bandwidth *W* is allocated to multiple UDs selecting the offloading computation mode, enabling parallel transmission. To ensure resource waste, channel bandwidth should only be allocated to UDs who select the edge computation mode. Thus, we have the following bandwidth constraints:

(2) $$\begin{array}{cc} \; & \sum_{i\in I_{p}}W_{i}\leq W,\forall I_{p} \end{array}$$

(3) $$\begin{array}{cc} \; & W_{i}\left\{\begin{array}{cc} =0, & d_{i}=0\\ >0, & d_{i}=1 \end{array}\right.,\forall i\in I \end{array}$$

Since air-to-ground communication only occurs when the UAV is in a hovering state, there is no Doppler frequency shift during communication due to the UAV’s high-speed movement [36]. Let $q_{i}=\left(x_{i},y_{i},0\right)$ represent the three-dimensional coordinates of UD *i*. When the UAV hovers over coverage circle *p* and provides task offloading services to UD *i*, the channel gain between them can be expressed as $h_{i}=\frac{g_{0}}{{\left(x_{p}-x_{i}\right)}^{2}+{\left(y_{p}-y_{i}\right)}^{2}+z^{2}}$, where $g_{0}$ represents the unit-distance channel gain at 1 m. The data transmission rate can be expressed as [9]:

(4) $$\begin{array}{cc} \; & r_{i}=W_{i}\cdot \log_{2}\left(1+\frac{P_{i}\cdot h_{i}}{N_{0}^{2}}\right) \end{array}$$

where $P_{i}$ denotes the transmission power of UD *i*, and $N_{0}$ represents the additive Gaussian white noise power. The delay of task $o_{i}$ offloaded to the UAV can be expressed as:

(5) $$\begin{array}{cc} \; & T_{i}^{o}=\frac{u_{i}}{r_{i}} \end{array}$$

While a more realistic approach in air-to-ground communications indeed involves a probabilistic channel model that accounts for both line-of-sight (LoS) and non-line-of-sight (NLoS) conditions, as noted in [5], our study simplifies the scenario by assuming communication occurs solely when the UAV is hovering. In this state, the relative positions of users and the UAV are considered static, allowing our simplified model to be adapted to probabilistic channels. This adaptation is achieved by applying the probabilistic channel formula, as detailed in [37], thus extending our model’s applicability to scenarios where both LoS and NLoS conditions coexist.

Assuming that tasks offloaded to the UAV can be executed in parallel, the UAV’s computational resources need to be allocated to multiple user tasks. Let $f^{edge}$ represent the computational frequency of the edge server and $f_{i}$ represent the computational frequency allocated to UD *i* by the edge server. Then, in the edge computation mode, the execution time of task $o_{i}$ on the UAV can be expressed as

(6) $$\begin{array}{cc} \; & T_{i}^{e}=\frac{c_{i}}{f_{i}} \end{array}$$

Similar to the bandwidth allocation Constraints (2) and (3), the allocation of UAV’s computational resources should also satisfy the following conditions:

(7) $$\begin{array}{cc} \; & \sum_{i\in I_{p}}f_{i}\leq f^{edge},\;\forall I_{p} \end{array}$$

(8) $$\begin{array}{cc} \; & f_{i}\left\{\begin{array}{cc} =0, & d_{i}=0\\ >0, & d_{i}=0 \end{array}\right.,\forall i\in I \end{array}$$

We consider a fixed hover time; that is, the UAV hovers in each area for the same duration $T^{h}$, and the time required for the UAV to fly from the previous area $w_{p-1}$ to the area $w_{p}$ is denoted as $T_{w_{p}}^{f}$. The total time required for the area $w_{p}$ to wait for the UAV to reach the center of the area can be expressed as

(9) $$\begin{array}{cc} T_{w_{p}}^{w} & =\sum_{j=1}^{p-1}\left(T_{w_{j}}^{f}+T^{h}\right)+T_{w_{p}}^{f} \end{array}$$

In the edge computation mode, the total delay incurred by user *i* in processing its computational task within coverage area $w_{p}$ can be expressed as

(10) $$T_{i}^{e}=T_{w_{p}}^{w}+T_{i}^{o}+T_{i}^{e},\forall i\in I_{w_{p}}$$

In addition, due to the usually negligible transmission time compared to task offloading and drone computation [38], the total latency incurred by user *i* in processing its computing task can be expressed as

(11) $$T_{i}=d_{i}\cdot T_{i}^{e}+\left(1-d_{i}\right)\cdot T_{i}^{l}$$

### 3.3. Problem Formulation

We aim to optimize the QoE of task offloading services in UAV-edge networks, a goal achieved by minimizing the sum of all user devices’ shrinkage rates. Similar to [16], the sum of system shrinkage rates can be represented as

(12) $$\begin{array}{cc} S & =\sum_{w_{p}\in w}\sum_{i\in I_{w_{p}}}S_{i}=\sum_{w_{p}\in w}\sum_{i\in I_{w_{p}}}\frac{T_{i}}{T_{i}^{l}} \end{array}$$

By jointly optimizing the partitioning strategy of coverage areas P, the UD’s computation mode selection $d=\{d_{i}\}$, uplink bandwidth $B=\{B_{i}\}$, and UAV’s computation resource allocation strategy $f=\{f_{i}\}$, the objective is to minimize the sum of all users’ shrinkage rates. It is noteworthy that the coverage strategy P here includes the coordinates of each coverage circle and the set of user devices it contains. Thus, the aforementioned problem of minimizing the sum of shrinkage rates can be formulated as

(13) $$\begin{array}{cc} \left(P1\right)\;\min_{P,d,B,f}\; & S\\ s.t.\; & \left(2),(3),(7),(8\right) \end{array}$$

(14) $$\begin{array}{cc} \; & d_{i}\in \left\{0,1\right\},\forall i\in I \end{array}$$

(15) $$\begin{array}{cc} \; & I_{p}\neq \emptyset ,\forall p\in P \end{array}$$

(16) $$\begin{array}{cc} \; & I_{p_{1}}\cap I_{p_{2}}=\emptyset ,\forall p_{1},p_{2}\in P,p_{1}\neq p_{2} \end{array}$$

(17) $$\begin{array}{cc} \; & I_{1}\cup I_{2}\cup \ldots \cup I_{P}=I \end{array}$$

(18) $$\begin{array}{cc} \; & T_{i}^{o}+T_{i}^{e}\leq T^{h},\forall i\in I \end{array}$$

Here, Constraints (2), (3), (7), and (8) ensure that the uplink bandwidth for task offloading and the UAV’s computation resources are allocated only to valid links, (15)–(17) restrict the feasibility of the coverage strategy, and (18) ensure that each device completes the task offloading within the hover time. Due to the involvement of binary Constraint (14) and a non-convex objective function, P1 is a coupled MINLP problem. Clearly, solving it directly is challenging.

## 4. Algorithm Design

**Figure 2:**
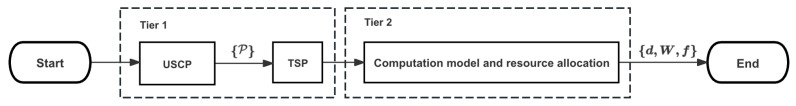
The solution flowchart of the global algorithm.

In this section, an efficient two-tier algorithm is proposed to address the formulated non-convex problem. Firstly, a polynomial-time complexity greedy algorithm based on the Welzl method [39] for the UAV set covering problem (USCP) is proposed to obtain a feasible P. Secondly, from the perspectives of algorithm complexity and solution quality, two different strategies are proposed in the second tier for computation mode selection and resource allocation problems. The process of the global algorithm is depicted in Figure 2.

### 4.1. UAV Set Covering Probelm

Since the user devices within a single coverage circle can concurrently receive task offloading services provided by the UAV, to enhance system efficiency, we aim to minimize the number of coverage areas, thus minimizing the time spent by UAVs in position shifting. This problem can be described as a fixed-radius circular coverage problem or an UAV set covering problem, which aims to cover all UDs within the system area using multiple circles with a fixed radius *r*, while minimizing the number of circles used. This is a typical NP-hard problem for which there is no polynomial-time exact algorithm. In this section, we propose the Welzl-based UAV set covering (WUSC) algorithm, which provides an approximate solution to the coverage circle set in polynomial time.

**Figure 3:**
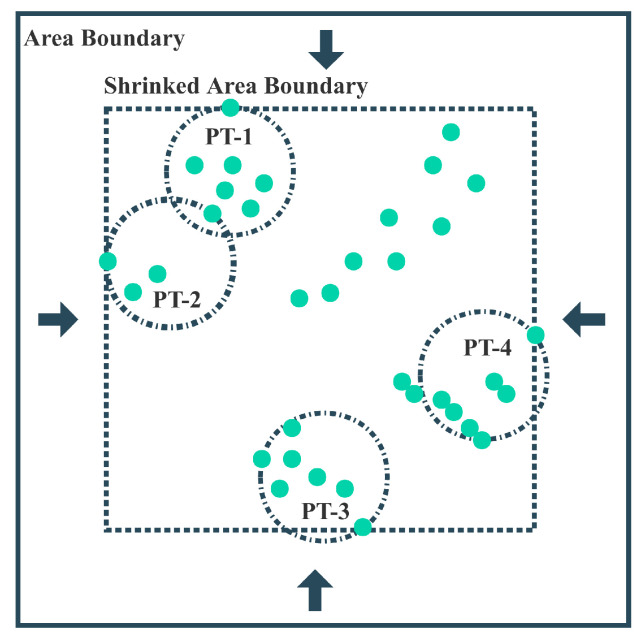
The WUSC algorithm for iteratively shrinking regions.

The Welzl algorithm is an incremental algorithm used to solve the minimum enclosing circle problem. It takes all points within a circular coverage area and returns the minimum radius of the circle. We consider iteratively invoking this algorithm to solve the fixed-radius circular coverage problem. As shown in Figure 3, the boundary of the system area is continuously reduced until there exists a device exactly on the boundary. Then, starting from a point *i* on the boundary and initializing a coverage area $I_{p}=\left\{i\right\}$, the device closest to this point within the region is added to the set $I_{p}$, and it is determined using the Welzl algorithm whether the set can be completely covered by a circle with a radius not exceeding *r*. This process is repeated until all user devices are included in some coverage set. Algorithm 1 describes the detailed procedure of the WUSC algorithm.
**Algorithm 1:** WUSC algorithm
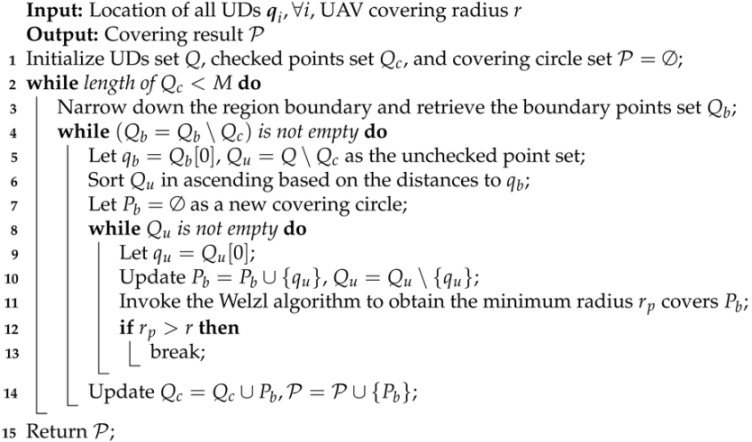


UAV trajectory planning is indeed a critical and challenging aspect of network optimization, with prevailing research typically employing SCA or DRL methods. However, these approaches encounter limitations in terms of computational complexity and generalizability, particularly for large-scale networks. To enhance solution efficiency, we adopt a pragmatic simplification by reducing UAV trajectory planning to a partition-based shortest path problem, as referenced in [40]. This approach enables a more tractable solution by decomposing the problem into smaller-scale TSP instances, as illustrated in Figure 2. Importantly, our algorithm retains the flexibility to incorporate more sophisticated methods like SCA and DRL for UAV path determination should the need for broader applicability arise.

Then, P1 can be reformulated as:

(19) $$\begin{array}{cc} \left(P2\right)\;\min_{d,W,f}\; & S\\ s.t.\; & \left(2),(3),(7),(8),(14),(18\right) \end{array}$$

Note that P2 is still an MINLP problem. However, since the hover time is constant, there is no coupling between multiple partitions, and thus, the problem can be divided into multiple smaller sub-problems for a parallel solution based on the partition dimension. We will present the algorithms for P2 as follows:

### 4.2. Computation Mode Selection and Resource Allocation

We can decompose P2 into *P* independent sub-problems in the following form according to the coverage areas:

(20) $$\begin{array}{cc} \left(P2.1\right)\;\min_{d_{p},W_{p},f_{p}}\; & \sum_{i\in I_{w_{p}}}\frac{d_{i}\cdot \left(T_{w_{p}}^{w}+T_{i}^{o}+T_{i}^{e}\right)+\left(1-d_{i}\right)\cdot T_{i}^{l}}{T_{i}^{l}}\\ s.t.\; & \left(2),(3),(7),(8),(14),(18\right) \end{array}$$

For problem P2.1, we will propose two different algorithms to solve it. The first is a low-complexity CD-based algorithm, suitable for smaller-scale IoT networks. The second is a joint optimization algorithm based on the ADMM technique, designed for larger-scale networks that require higher solution precision.

#### 4.2.1. Alternating Optimization Using CD Method

In this section, we consider alternating iterative optimization of discrete and continuous variables. Given the computation mode d, let $I_{p}\left(0\right)$ represent the set of UDs choosing local computation mode in coverage area *p* and $I_{p}\left(1\right)$ represent the set of users choosing edge computation mode. Then, P2.1 can be equivalently transformed into the following form:

(21) $$\begin{array}{cc} \left(P2.1^{\prime }\right)\;\min_{W_{p},f_{p}}\; & \sum_{i\in I_{p}\left(1\right)}\frac{T_{i}^{o}+T_{i}^{e}}{T_{i}^{l}}\\ s.t.\; & \left(2),(7),(18\right) \end{array}$$

(22) $$\begin{array}{cc} \; & W_{i},f_{i}>0,\forall i\in I_{p}\left(1\right) \end{array}$$

P2.1’ is a convex problem, which can obtain the optimal closed-form solution for the problem using the KKT conditions. By introducing Lagrange multipliers $\lambda =\{\lambda_{i},\forall i\in I\},$$\nu_{1},\nu_{2}$ for the inequality Constraints (2), (7) and (18), we can obtain the Lagrangian function as

(23) $$\begin{array}{cc} L(W_{p},f_{p}; & \lambda_{p},\nu_{1},\nu_{2})=\sum_{i\in I_{p}\left(1\right)}\left\{\frac{u_{i}}{W_{i}\cdot \log_{2}\left(1+\frac{P_{i}\cdot h_{i}}{N_{0}^{2}}\right)\cdot T_{i}^{l}}+\frac{c_{i}}{f_{i}\cdot T_{i}^{l}}+\lambda_{i}\cdot \left[-T^{h}+\frac{c_{i}}{f_{i}}\right.\right.\\ \; & \left.\left.+\frac{u_{i}}{W_{i}\cdot \log_{2}\left(1+\frac{P_{i}\cdot h_{i}}{N_{0}^{2}}\right)}\right]\right\}+\nu_{1}\cdot \left(\sum_{i\in I_{p}\left(1\right)}W_{i}-W\right)+\nu_{2}\cdot \left(\sum_{i\in I_{p}\left(1\right)}f_{i}-f^{edge}\right) \end{array}$$

The corresponding KKT conditions can be derived as

(24) $$\begin{array}{c} \left\{\begin{array}{cc} 1+\lambda_{i}-\nu_{1}\cdot \frac{W_{i}^{2}\cdot \log_{2}\left(1+\frac{P_{i}\cdot h_{i}}{N_{0}^{2}}\right)\cdot T_{i}^{l}}{u_{i}}=0,\forall i\in I_{p}\left(1\right) & \left(a\right)\\ 1+\lambda_{i}-\nu_{2}\cdot \frac{f_{i}^{2}\cdot T_{i}^{l}}{c_{i}}=0,\forall i\in I_{p}\left(1\right) & \left(b\right)\\ \nu_{1}\cdot \left(\sum_{i\in I_{p}\left(1\right)}W_{i}-W\right)=0 & \left(c\right)\\ \nu_{2}\cdot \left(\sum_{i\in I_{p}\left(1\right)}f_{i}-f^{edge}\right)=0 & \left(d\right)\\ \sum_{i\in I_{p}\left(1\right)}W_{i}-W\leq 0 & \left(e\right)\\ \sum_{i\in I_{p}\left(1\right)}f_{i}-f^{edge}\leq 0 & \left(f\right)\\ \lambda_{i},\nu_{1},\nu_{2}\geq 0 & \left(g\right)\\ \lambda_{i}\cdot \left[\frac{u_{i}}{W_{i}\cdot \log_{2}\left(1+\frac{P_{i}\cdot h_{i}}{N_{0}^{2}}\right)}+\frac{c_{i}}{f_{i}}-T^{h}\right]=0,\forall i\in I_{p}\left(1\right) & \left(h\right)\\ \frac{u_{i}}{W_{i}\cdot \log_{2}\left(1+\frac{P_{i}\cdot h_{i}}{N_{0}^{2}}\right)}+\frac{c_{i}}{f_{i}}-T^{h}\leq 0,\forall i\in I_{p}\left(1\right) & \left(i\right) \end{array}\right. \end{array}$$

Based on the KKT system of Equation (24), we can derive the following lemma:

**Lemma 1.**  *When the optimal solution of P2.1’ is obtained, the resources are fully allocated to the users, i.e., $\sum_{i\in I_{p}\left(1\right)}W_{i}=W,\sum_{i\in I_{p}\left(1\right)}f_{i}=f^{edge}$ hold strictly.*

**Proof.**  From (a), (b), (c), (d), (g), it can be inferred that $\nu_{1},\nu_{2}>0$. Substituting (c) and (d) into the equations yields $\sum_{i\in I_{p}\left(1\right)}W_{i}=W$ and $\sum_{i\in I_{p}\left(1\right)}f_{i}=f^{edge}$, which means that the bandwidth and computational resources should be allocated entirely to the users when the optimal solution is achieved.    □

We refer to the resource allocation strategy obtained without considering Constraints (18) as the optimal solution $S^{OPT}$ and the optimal solution of P2.1’ as a suboptimal solution $S^{*}$. Based on whether $S^{OPT}=S^{*}$, we can analyze P2.1’ in the two different cases as follows:

(1) $S^{OPT}=S^{*}$, meaning that there are no UDs violating the hovering constraint under $S^{*}$. In this case, we have the following lemma for this optimal solution.

**Lemma 2.**  When $S^{OPT}=S^{*}$ holds, both $W_{p}^{*}$ and $f_{p}^{*}$ have corresponding closed-form solutions.

**Proof.**  We have $\lambda_{p}=0$ when hovering constraints are not in effect. Based on (a) and (b), we have $W_{i}^{2}=\frac{u_{i}}{\nu_{1}\cdot \log_{2}\left(1+\frac{P_{i}\cdot h_{i}}{N_{0}^{2}}\right)\cdot T_{i}^{l}}$ and $f_{i}^{2}=\frac{c_{i}}{\nu_{2}\cdot T_{i}^{l}}$. Then we have $\sqrt{\nu_{1}}=\frac{\sum_{i\in I_{p}\left(1\right)}\sqrt{\frac{u_{i}}{\log_{2}\left(1+\frac{P_{i}\cdot h_{i}}{N_{0}^{2}}\right)\cdot T_{i}^{l}}}}{W}$ and $\sqrt{\nu_{2}}=\frac{\sum_{i\in I_{p}\left(1\right)}\sqrt{\frac{c_{i}}{T_{i}^{l}}}}{f^{edge}}$. Finally, substituting $\nu_{1}$ and $\nu_{2}$ into (a) and (b) yields the following closed-form solutions for $W_{i},f_{i},\forall i\in I_{p}\left(1\right)$:

(25) $$\begin{array}{c} W_{i}=\frac{\sqrt{\frac{u_{i}}{\log_{2}\left(1+\frac{P_{i}\cdot h_{i}}{N_{0}^{2}}\right)\cdot T_{i}^{l}}}}{\sum_{j\in I_{p}\left(1\right)}\sqrt{\frac{u_{j}}{\log_{2}\left(1+\frac{P_{j}\cdot h_{j}}{N_{0}^{2}}\right)\cdot T_{j}^{l}}}}\cdot W,\;f_{i}=\frac{\sqrt{\frac{c_{i}}{T_{i}^{l}}}}{\sum_{j\in I_{p}\left(1\right)}\sqrt{\frac{c_{j}}{T_{j}^{l}}}}\cdot f^{edge} \end{array}$$

   □

(2) $S^{OPT}\neq S^{*}$, which means that some UDs violate the hover constraint under $S^{OPT}$. If the $S^{*}$ has a solution, it indicates that the suboptimal strategy assigns additional bandwidth or computation resources to those UDs that exceed the hover time, allowing them to complete the computation within $T^{h}$. However, this approach simultaneously increases the time consumed for other non-violating devices. Let $I_{p}^{A}=\{i|i\in I_{p}\left(1\right),$$T_{i}^{o}+T_{i}^{e}>T^{h}\}$ denote the set of UDs that choose the edge computing mode and violate the hover constraint, and $I_{p}^{B}=I_{p}\left(1\right)\setminus I_{p}^{A}$ represents the others. According to (24), a complex system of equations with $\left(3|I_{p}\left(1\right)|+2\right)$ variables and equalities can be derived. Enumerating $2^{I}$ possible closed-form solutions is impractical. Moreover, if the $S^{*}$ has no solution, it implies the bandwidth and computation resource of the UAV are insufficient to meet the computational demands of all selected offloading UDs within the given hover time $T^{h}$. Therefore, these two parts, {d} and {W,f}, cannot be fully decoupled by the CD method.

In summary, we can obtain the optimal {W,f} in O(1) using (25) for the first type of problem. For the second type, the corresponding KKT equations either have no solution or are challenging to solve directly. To minimize coupling and simplify the solution of the KKT equations, a greedy strategy can be adopted by converting the second type problem approximately to the first type. Additionally, for the computation mode {d}, we consider obtaining a locally optimal solution through a simple one-dimensional linear search. For convenience, let $d_{p}=\left(d_{1},d_{2},\ldots ,d_{\left|p\right|}\right)$ represent the computation modes of all UDs within coverage area *p*. At the beginning of the (l−1)-th iteration, the initial value is defined as $d_{p}^{l-1}$. Each user attempts to change their computation mode based on $d_{p}^{l-1}$:

(26) $$\begin{array}{c} d_{p}^{l-1}\left(i\right)=\left(d_{1}^{l-1},\ldots ,d_{i-1}^{l-1},⊕\left(d_{i}^{l-1}\right),d_{i+1}^{l-1},\ldots ,d_{\left|p\right|}^{l-1}\right) \end{array}$$

where ⊕(·) denotes the negation operation for binary variables, e.g., ⊕(1)=0, ⊕(0)=1. This generates |p| different computation modes, and the one with the smallest shrinkage rate is selected as the return value for the (l−1)-th iteration. A locally optimal solution is achieved when no user can further reduce the global shrinkage rate by changing their computation mode. Algorithm 2 describes the detailed procedure of the CD-based greedy (CD-Greedy) algorithm for P2.1.
**Algorithm 2:** CD-Greedy Algorithm for P2.1
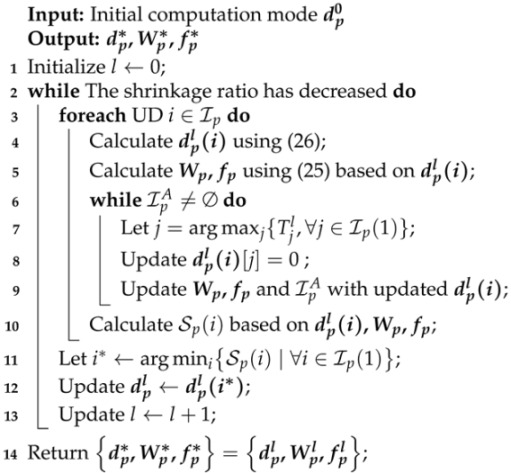


#### 4.2.2. Joint Optimization Using ADMM Method

The main advantage of the CD-based algorithm lies in its simplicity and relatively high efficiency due to the closed-form resource allocation strategy. However, alternating optimization is prone to falling into local optima, and the one-dimensional search causes the number of iterations required for convergence to increase with the network scale. In this section, we will propose an ADMM-based algorithm to jointly optimize computation mode and resource allocation. The main idea is to decompose the coupled problem P2.1 into $|I_{p}|$ parallel small-scale MINLP problems. First, to eliminate the coupled inequality constraints, we introduce auxiliary variables to reformulate P2.1 into the following equivalent form:

(27) $$\begin{array}{cc} \left(P2.2\right)\;\min_{d_{p},W_{p},f_{p},x_{p},y_{p}}\; & \sum_{i\in I_{p}}q\left(d_{i},W_{i},f_{i}\right)+g\left(x_{p},y_{p}\right)\\ s.t.\; & \left(14),(22\right) \end{array}$$

(28) $$\begin{array}{cc} \; & x_{i},y_{i}\geq 0,\forall i\in I_{p} \end{array}$$

(29) $$\begin{array}{cc} \; & x_{i}=W_{i},y_{i}=f_{i},\forall i\in I_{p} \end{array}$$

(30) $$\begin{array}{cc} \; & \sum_{i\in I_{p}}x_{i}\leq W,\sum_{i\in I_{p}}y_{i}\leq f^{edge} \end{array}$$

(31) $$\begin{array}{cc} \; & \frac{u_{i}}{x_{i}\cdot \log_{2}\left(1+P_{i}\cdot h_{i}/N_{0}^{2}\right)}+\frac{c_{i}}{y_{i}}\leq T^{h},\forall i\in I_{p} \end{array}$$

where $q(d_{i},W_{i},f_{i})$ is the simplified form of the original objective function:

(32) $$\begin{array}{c} q\left(d_{i},W_{i},f_{i}\right)=\frac{d_{i}\cdot \left(T_{w_{p}}^{w}+T_{i}^{o}+T_{i}^{e}\right)+\left(1-d_{i}\right)\cdot T_{i}^{l}}{T_{i}^{l}} \end{array}$$

$g(x_{p},y_{p})$ is the equivalent form of the inequality constraints in the original problem:

(33) $$\begin{array}{cc} g(x_{p},y_{p}) & =\left\{\begin{array}{cc} 0 & \mathrm{if}\;\left(x_{p},y_{p}\right)\in G_{p}\\ +\infty & otherwise \end{array}\right. \end{array}$$

where $G_{p}$ denotes the feasible set of $g(x_{p},y_{p})$:

(34) $$G_{p}=\left\{\left(x_{p},y_{p}\right)|\sum_{i\in I_{p}}x_{i}\leq W,\sum_{i\in I_{p}}y_{i}\leq f^{edge};\frac{u_{i}}{x_{i}\cdot \log_{2}\left(1+P_{i}\cdot h_{i}/N_{0}^{2}\right)}+\frac{c_{i}}{y_{i}}\leq T^{h},\forall i\in I_{p}\right\}$$

Problem P2.2 can be effectively decomposed using the ADMM method to find the optimal solution to the dual problem. By introducing Lagrange multipliers for the equality Constraint (29), we can represent the augmented Lagrangian function of P2.2 as

(35) $$\begin{array}{cc} L(u,v,\theta ) & =\sum_{i\in I_{p}}q\left(u\right)+g\left(v\right)+\alpha_{i}\cdot \left(W_{i}-x_{i}\right)+\beta_{i}\cdot \left(f_{i}-y_{i}\right)\\ \; & \;\;\;-\frac{c}{2}\cdot \sum_{i\in I_{p}}{\left(W_{i}-x_{i}\right)}^{2}-\frac{c}{2}\cdot \sum_{i\in I_{p}}{\left(f_{i}-y_{i}\right)}^{2} \end{array}$$

where $u=\{d_{p},W_{p},f_{p}\}$, $v=\{x_{p},y_{p}\}$, $\theta =\{\alpha_{p},\beta_{p}\}$, and c>0 represents the step size. The corresponding dual problem can be formalized as

(36) $$\begin{array}{c} \left(P2.3\right)\;\max_{\theta }\;d\left(\theta \right) \end{array}$$

where $d\left(\theta \right)=\min_{u,v}\left\{L(u,v,\theta )|u\in U,v\in V\right\}$ represents the Lagrangian dual function of L, $U=\{\left(d_{p},W_{p},f_{p}\right)|W_{i},f_{i}\geq 0,d_{i}\in \left\{0,1\right\},\forall i\in I_{p}\}$, $V=\{\left(x_{p},y_{p}\right)|x_{i},y_{i}\geq 0,\forall i\in I_{p}\}$.

The ADMM method solves the dual problem P2.3 by iteratively optimizing {u,v,θ}. Let the solution obtained at the *l*-th iteration be $\left\{u^{l},v^{l},\theta^{l}\right\}$. The operations required in the (l+1)-th iteration can be described as the following three steps:

Step 1: Given $\left\{v^{l},\theta^{l}\right\}$, minimize L by finding suitable $\left\{u^{l}\right\}$.
  (37) $$\begin{array}{c} u^{l+1}=\arg\min_{u}L\left(u,v^{l},\theta^{l}\right) \end{array}$$
  Since there is no coupling among multiple users, (37) can be decomposed into $|I_{p}|$ identical subproblems solved in parallel. Each subproblem can be formulated as
  (38) $$\begin{array}{cc} u_{i}^{l+1} & =\arg\min_{u\in U}q\left(d_{i},W_{i},f_{i}\right)+\alpha_{i}\cdot \left(W_{i}-x_{i}\right)+\beta_{i}\cdot \left(f_{i}-y_{i}\right)-\frac{c}{2}\cdot \left[{\left(W_{i}-x_{i}\right)}^{2}+{\left(f_{i}-y_{i}\right)}^{2}\right] \end{array}$$
  When $d_{i}=0$, we have $B_{i}=f_{i}=0$. In this case, $u_{i}^{l+1}$ can be directly computed using (38). When $d_{i}=1$, (38) becomes a standard convex optimization problem and yields a closed-form optimal solution. For example, by separating $W_{i}$ and $f_{i}$ into two independent parts, setting the first derivative to zero, and combining with the Cardano formula, we can obtain a unique optimal solution. Therefore, we consider the two possible values of $d_{i}$ separately. After obtaining the corresponding $W_{i}$ and $f_{i}$, they are substituted into (38), and the smaller value is chosen as the optimal solution for $u_{i}^{l+1}$. Note that the time complexity for solving each subproblem $u_{i}^{l+1}$ is O(1).
Step 2: Given $\left\{u^{l+1},\theta^{l}\right\}$, minimize L by finding suitable $\left\{v^{l+1}\right\}$.
  From (33), it is evident that to ensure the problem’s solvability, it is necessary to ensure $v^{l+1}\in G_{p}$. Since the offloading decisions are known, we only need to consider the auxiliary variables corresponding to the edge computing mode UDs. The solution for $v^{l+1}$ can be formulated as a convex problem in the following form:
  (39) $$\begin{array}{cc} \left(P2.4\right)\;\arg\min_{v}\; & \sum_{i\in I_{p}\left(1\right)}\alpha_{i}\cdot \left(W_{i}-x_{i}\right)+\beta_{i}\cdot \left(f_{i}-y_{i}\right)-\frac{c}{2}\cdot \left[{\left(W_{i}-x_{i}\right)}^{2}+{\left(f_{i}-y_{i}\right)}^{2}\right]\\ s.t.\; & \left(28),(29),(30),(31\right) \end{array}$$
  To obtain the optimal solution to P2.4, we consider using convex optimization solver, e.g., CPLEX, CVX. Solvers based on standard convex optimization methods like interior point methods ensure that the obtained solution is the optimal solution, thereby preventing the problem P2.4 from being infeasible due to g(v) taking on positive infinity.
Step 3: Given $\left\{u^{l+1},v^{l+1}\right\}$, minimize L by finding suitable {θ}.
  This can be achieved by updating the Lagrange multipliers $\theta_{m}^{l}$ according to the following rules:
  (40) $$\begin{array}{cc} \; & \alpha_{i}^{l+1}=\alpha_{i}^{l}-c\cdot \left(W_{i}^{l+1}-x_{i}^{l+1}\right) \end{array}$$
  (41) $$\begin{array}{cc} \; & \beta_{i}^{l+1}=\beta_{i}^{l}-c\cdot \left(f_{i}^{l+1}-y_{i}^{l+1}\right) \end{array}$$
  Obviously, the time complexity for updating the Lagrange multipliers is O(I).
  Repeat the above three steps until the absolute errors of u and v reach the desired accuracy. The absolute errors of both can be measured by the following equation:
  (42) $$\begin{array}{c} \sum_{i\in I_{p}}\left(|W_{i}^{l}-x_{i}^{l}\left|+\right|f_{i}^{l}-y_{i}^{l}|\right)\leq 2\delta \end{array}$$
  where δ is a predefined error bound, typically a very small positive number. Due to the presence of duality gap for P2.1, the ADMM algorithm may not converge to the optimal solution. Therefore, when the algorithm terminates, the dual optimal solution $\left\{d^{l},W^{l},f^{l}\right\}$ is an approximate solution, and its performance gap will be evaluated through simulation experiments. Algorithm 3 describes the detailed procedure of the ADMM-based joint optimization algorithm for P2.1.

**Algorithm 3:** ADMM-based Algorithm for P2.1
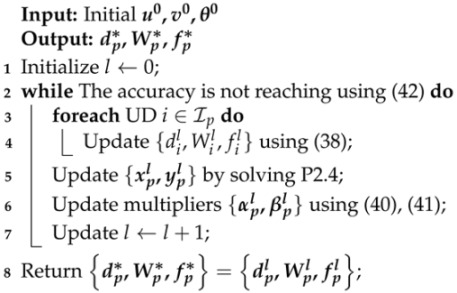


### 4.3. Computational Complexity Analysis

The WUSC algorithm consists of nested three-level loops, where the outer loop is used to check if there are any uncovered UDs, and the remaining two loops iterate over the boundary point set and attempt to construct a new coverage area starting from each boundary point. The time complexity of implementing the minimum circle covering of *n* vertices using the Welzl algorithm is O(n), thus the time complexity of generating a coverage area in each iteration of the middle loop is $O(|I_{p}{|}^{2})$. In addition, considering that WUSC employs sorting, the overall time complexity is $O(I^{2}\cdot \log\left(I\right))$.

Let $L_{CD}$ denote the number of iterations. The time complexity of CD-based greedy algorithm can be expressed as $O\left(L_{CD}\cdot \sum_{i\in I}\left|I_{p}\left(i\right)\right|\right)$ = $O\left(L_{CD}\cdot \sum_{p\in P}{\left|I_{p}\right|}^{2}\right)\leq$$O\left(L\cdot I^{2}\right)$, where $I_{p}\left(i\right)$ represents the partition where user *i* located. However, due to the computation modes searching along only one dimension for each iteration, in most cases, removing the UD with the maximum timeout only needs one. Therefore, the average time complexity is closer to $O(L_{CD}\cdot I)$.

The time complexity of the ADMM method is mainly determined by Step 2. For example, using an interior point method would result in a high time complexity of step 2 of $\sum_{p\in P}O(|I_{p}{|}^{3.5})\leq O\left(I^{3.5}\right)$. The total computational complexity can be given by $O(L_{ADMM}\cdot I^{3.5})$.

## 5. Performance Evaluation

### 5.1. Experimental Setup

In this section, we evaluate the performance of two algorithms through numerical experiments. The system area is a square with a side length of 1 km, containing a UAV carrying computing equipment to provide task offloading services for all UDs. We assume all UDs are homogeneous, with the same local computing frequency and transmission power; $f_{i}^{l}=100$ MHz, $p_{i}=1$ W, ∀i∈I. For the UAV, similar to [16], we set $f^{edge}=10$ GHz. Table 1 presents the values of system parameters.

### 5.2. Alternative Solutions

We compare our proposed algorithms—WUSC, CD-Greedy, and ADMM—with a selection of benchmark algorithms from recent literature, ensuring they are state-of-the-art and relevant to our study. Additionally, we incorporate classic algorithms such as LOCAL, EDGE, and Random to provide a comprehensive performance baseline.

- CD-OPT [41]: Similar in concept to CD-Greedy, CD-OPT employs a convex optimization solver to address problem P2.1’. Common in studies with a modest user scale, CD-OPT is a conventional choice for solving convex subproblems.
- SCA [42]: Widely used for joint optimization, SCA approximates non-convex objectives linearly for solver-based resolution. Prior work [16] has demonstrated the shrinkage ratio optimization capability of SCA; here, we focus on time consumption for comparison. For integer-variable problems, SCA relaxes offloading decisions and incorporates a penalty term to enforce binary outcomes.
- LOCAL: Represents a baseline where all UDs opt for local computation.
- EDGE: Unlike a complete offloading strategy, EDGE iteratively reassigns the UD with the highest shrinkage ratio to local computation until no UD exceeds a shrinkage ratio of 1.
- Random: Assigns computation modes to UDs randomly. If the resulting shrinkage rate is better than the previous iteration (starting with all local modes), it updates the offloading strategy accordingly.

### 5.3. Experiment Results

This section will demonstrate the effectiveness of the proposed algorithms through experimental results. The outcomes of the partition coverage algorithm are presented in the Section 5.3.1. The Section 5.3.2 analyzes the performance of the second-tier solution approach based on the experimental results of the global algorithm.

#### 5.3.1. Experiment Results for Partition Coverage Algorithm

We employ KMeans as a benchmark to evaluate the performance of our WUSC algorithm, a prevalent method in UAV-MEC systems for grouping UDs based on their geographic distribution. Our comparison specifically examines the efficiency of both algorithms in partitioning large-scale networks, with a key focus on how the number of required partitions correlates with the scale of UDs.

**Figure 4:**
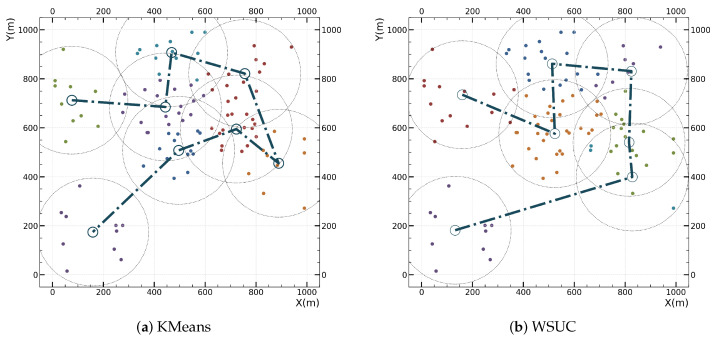
Comparison of covering and trajectory results between different covering methods. The dot-dash line represents the trajectory of the UAV based on the shortest path over the coverage area.

Figure 4 shows the results of performing fixed-radius circular coverage in the system area with 100 UDs using KMeans and WUSC algorithms, as well as the shortest path for the UAV departing from the specified coverage area. KMeans is a commonly used clustering algorithm in machine learning. The test results indicate that WUSC requires fewer coverage circles than KMeans, which to some extent reduces the time the UAV spends on location movement, thereby improving the utilization of bandwidth and computing resources. Experimental results show that, compared to the KMeans method, WUSC reduces the time the UAV spends on location movement by 10%.

**Figure 5:**
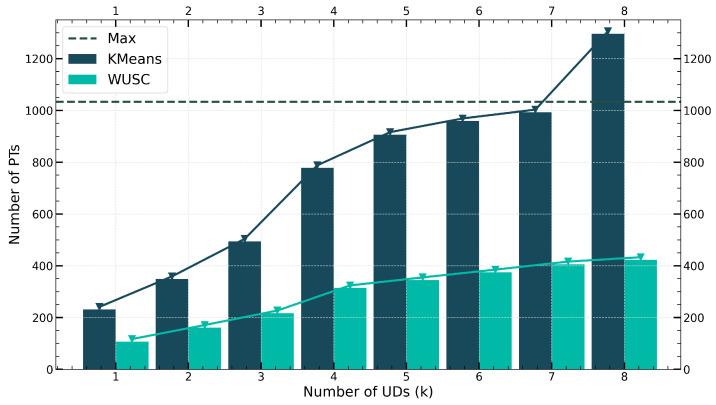
Number of partitions calculated by different UAV set covering algorithms.

Figure 5 depicts the area coverage results for different numbers of UDs in a larger area (10×10 km^2^). As the total number of UDs increases, the number of coverage circles required by WUSC slowly rises, while KMeans exceeds the maximum number of circles required to cover the entire area at M=8000. Compared to the KMeans method, WUSC requires approximately 60–70% fewer circles to cover the same set of user points, indicating that in large-scale fixed-radius circular coverage problems, the solution provided by WUSC is significantly superior to clustering methods.

#### 5.3.2. Experiment Results for Resource Allocation Algorithms

**Figure 6:**
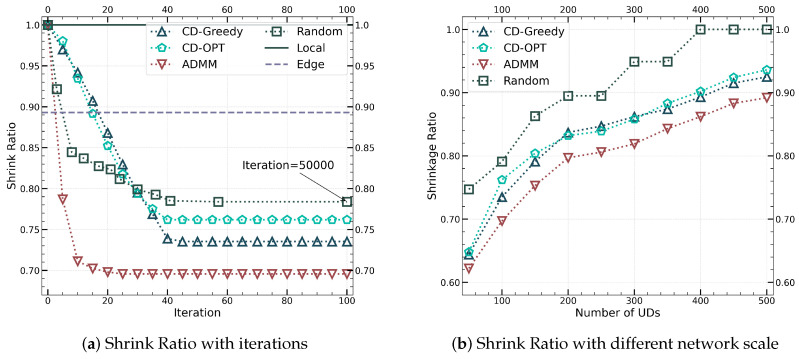
Comparison of shrink ratio between iterations and network scale.

Figure 6 illustrates the trend of shrinkage ratio for different algorithms, where Figure 6a represents the curve of shrinkage ratio converging with the increase of iteration numbers in a network with 100 UDs. Figure 6b shows the trend of shrinkage ratio changes for different algorithms as the network scale increases. It can be observed from Figure 6a that CD-Greedy and ADMM methods can both converge stably to better results than CD-OPT after nearly forty iterations. Our CD-Greedy algorithm achieves an optimal reduction of 5% in the shrinkage ratio compared to CD-OPT. Furthermore, the ADMM method outperforms CD-OPT by approximately 10% in reducing the shrinkage ratio. Additionally, ADMM demonstrates a significant reduction in computational effort by requiring only half the number of iterations for convergence compared to the alternating optimization method based on CD. When compared to benchmark algorithms such as Random, Local, and Edge, all three of our proposed algorithms exhibit excellent convergence properties. Notably, the jointly optimized ADMM method shows a nearly 5% improvement in shrinkage ratio over CD-Greedy. As illustrated in Figure 6b, once the number of edge nodes surpasses 200, the convergence results of CD-Greedy and CD-OPT become virtually identical, whereas ADMM consistently realizes nearly a 5% enhancement in shrinkage ratio performance relative to CD-based methods.

**Figure 7:**
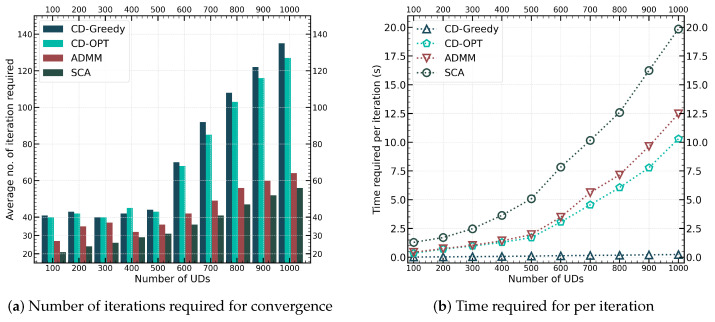
Comparison of convergence performance between different network scale.

In Figure 7, we examine the convergence performance of the CD-Greedy, CD-OPT, SCA, and ADMM methods. Figure 7a shows the variation of the number of iterations required for convergence of these algorithms as the number of devices increases from 100 to 1000. When the total number of UDs increases from 100 to 500, the computing capability of the UAV remains constant. When the number of UDs increases from 500 to 1000, we adjust the computing performance of the UAV to 10 GHz, 20 GHz …, respectively. Figure 7 shows that when the computing resources of the UAV are limited, the number of iterations required for convergence of both methods remains stable, essentially equal to the total number of devices finally offloaded. However, as the computing resources of the UAV increase, more UDs are eventually selected for offloading, leading to a faster increase in the number of iterations required for convergence in CD-Greedy and CD-OPT. In comparison, the number of iterations for the SCA and ADMM methods increases more slowly, basically maintaining around 40% of the CD-based methods. This highlights the advantage of joint optimization algorithms over alternating optimization algorithms.

Figure 7b illustrates the trend of the average execution time per iteration of four methods with the increase in network size. It can be seen that the execution time of the convex optimization-based methods (CD-OPT, SCA, and ADMM) is more obviously affected by the problem size. However, since SCA and ADMM require fewer iterations to converge, the overall convergence time is shorter compared to CD-OPT. The time required for a single iteration of the ADMM method is about 30% lower than that of the SCA method. Because the SCA that jointly optimizes integer and continuous variables has *M* more decision variables than solving P2.4, and the form of the optimization objective is more complex. Although the SCA method requires 15% fewer iterations compared to ADMM, the increased time for each iteration results in the overall runtime of the ADMM method being lower than SCA. It is worth noting that the CD-Greedy method reduces the time consumption per iteration by three orders of magnitude compared to the other methods, consistently remaining at the millisecond level. This demonstrates the performance advantage of CD-Greedy in large-scale edge networks.

**Figure 8:**
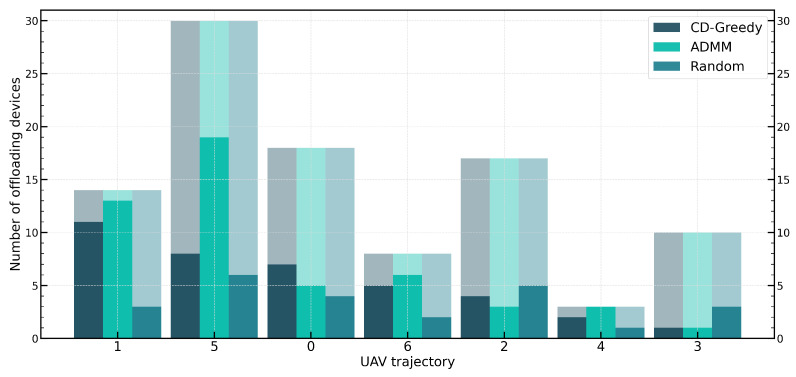
Comparison of offloading UDs in each covering circle.

Figure 8 illustrates the proportion of UDs offloaded in different coverage areas, with the coverage areas sorted according to the UAV’s trajectory. The results of CD-OPT are similar to those of CD-Greedy, so they are not shown in the figure. From the graph, it can be observed that both CD-Greedy and ADMM tend to select coverage areas near the beginning of the trajectory for offloading. This is because the fixed hovering time ignores the decision of coverage areas’ influence on subsequent areas. Moreover, since coverage areas near the beginning of the trajectory typically have shorter waiting times, offloading more UDs in these areas will result in lower compression ratios. Additionally, due to limited resources in the system, the probability of selecting coverage areas for offloading decreases further when the number of devices selected for offloading reaches a certain threshold. Furthermore, if multiple consecutive service periods are considered, the coverage area at the end of the previous period will become the starting point for the UAV in the next period, thereby achieving relative fairness among multiple coverage areas.

## 6. Conclusions and Future Work

In this paper, we investigate the minimization of the shrinkage ratio in large-scale UAV-MEC networks. To achieve efficient optimization strategies in large-scale networks, we utilize a partitioning concept combined with a fixed hovering time to decouple interactions between multiple regions, resulting in two low-complexity algorithms. The CD-Greedy algorithm is simple to implement, converges stably, and is widely applicable to various mixed-decision, efficient optimization scenarios. The ADMM method, while achieving better optimization results by jointly optimizing integer and continuous variables with fewer iterations, has a longer execution time due to convex optimization, making it suitable for scenarios where high-quality solutions are desired and timeliness is not critical.

In our future endeavors, we are set to advance our research in the following directions: (1) Enhanced UAV Trajectory Planning: We will refine our UAV trajectory planning by leveraging partition-based approaches in conjunction with the traveling salesman problem, aiming to significantly elevate the optimization efficiency of our algorithms; (2) Inclusion of Energy Consumption Constraints: We intend to integrate energy consumption constraints into our model to address the dynamic nature of UAV flight trajectories, ensuring a more realistic and sustainable approach to energy management during flight operations; (3) Adaptive Online Task Offloading: Furthermore, we will explore the implementation of online task offloading in fluctuating environments using Deep Reinforcement Learning methods, with the goal of achieving a more responsive and efficient allocation of resources in the face of network uncertainties.

## Figures and Tables

**Table 1:** Parameter Settings for Simulations.

Parameters	Value
Number of UEs *I*	{100, 200, 300, …, 1000}
Height of UAV	50 m
Data size of tasks $u_{i}$	[10, 50] MB
Number of CPU cycles	{100, 200, 300} cycles/bit
Transmit power of UEs $p_{i}$	1 W
Computation resource of UEs $f_{i}^{l}$	100 Megacycles
Computation resource of UAV $f^{edge}$	10 Gigacycles
Flying speed of UAV $v^{max}$	20 m/s
Channel Bandwidth *W*	10 MHz
Path loss exponent $g_{0}$	−50 dBm
Noise power $N_{0}^{2}$	−100 dBm

## Data Availability

The main data are included in the article, and detailed numerical experimental data are available on request from the corresponding author.
